# Primary Neuroendocrine Carcinoma of Ocular Adnexa

**DOI:** 10.1155/2013/281351

**Published:** 2013-11-03

**Authors:** Daisuke Yamanouchi, Toshiyuki Oshitari, Yosuke Nakamura, Jiro Yotsukura, Kaoru Asanagi, Takayuki Baba, Tomohiro Nizawa, Takashi Kishimoto, Yoko Yonemori, Satoshi Ota, Shuichi Yamamoto

**Affiliations:** ^1^Department of Ophthalmology and Visual Science, Chiba University Graduate School of Medicine, Inohana 1-8-1, Chuo-ku, Chiba 260-8670, Japan; ^2^Department of Molecular Pathology, Chiba University Graduate School of Medicine, Inohana 1-8-1, Chuo-ku, Chiba 260-8670, Japan; ^3^Department of Diagnostic Pathology, Chiba University Graduate School of Medicine, Inohana 1-8-1, Chuo-ku, Chiba 260-8670, Japan

## Abstract

We present our findings in a case of primary neuroendocrine carcinoma (NEC) of the lacrimal gland and a case of primary Merkel cell carcinoma (MCC) of the eyelid. An 86-year-old man noticed a swelling of the left upper eyelid three months earlier. We performed excision biopsy and histopathological examination indicated that he had a primary NEC of the left lacrimal gland. He underwent chemotherapy followed by excision including the clinically visible margins and 50 Gy radiotherapy of the surgical margins. He had neither recurrence nor metastasis for 6 months since the last radiotherapy. An 80-year-old man noticed a nodule in the right upper eyelid and was referred to our hospital because the size was increasing rapidly. A complete surgical excision of the margins of the tumor was performed with histopathological confirmation of negative margins. The final diagnosis was a primary MCC of the right upper eyelid. After surgery, he underwent 50 Gy radiotherapy on the neck to prevent metastasis. No recurrence or metastasis was found for two years. Although primary NEC of the ocular adnexa is extremely rare, the tumor has high malignancy and readily metastasizes. Thus, combined therapy including surgery, radiotherapy, and/or chemotherapy is needed for complete management of NEC.

## 1. Introduction

Primary small cell neuroendocrine carcinomas (NECs) of extrapulmonary sites are extremely rare but are very important to diagnose because they are very aggressive [1]. Only one case of sinonasal small cell neuroendocrine carcinoma with orbital invasion has been reported, and the patient died in the second year of treatment [2]. Merkel cell carcinoma is an aggressive cutaneous NEC without hormonal syndromes, and 5 to 10% of Merkel cell carcinomas occur in the eyelids probably because the eyelids are frequently exposed to sunlight [3, 4]. Merkel cell carcinomas are also very rare and the estimated incidence is about 1/500,000 for Caucasians [4]. Only two cases of primary Merkel cell carcinoma of the lacrimal gland have been reported [5, 6]. 

NECs have a poor prognosis, and no standard treatment has been established. We present our findings in a case of primary small cell NEC of the lacrimal gland and a case of primary Merkel cell carcinoma of the upper eyelid. We also discuss the treatment for primary NECs of the ocular adnexa.

## 2. Case Reports

### 2.1. Case  1

**Figure 1:**
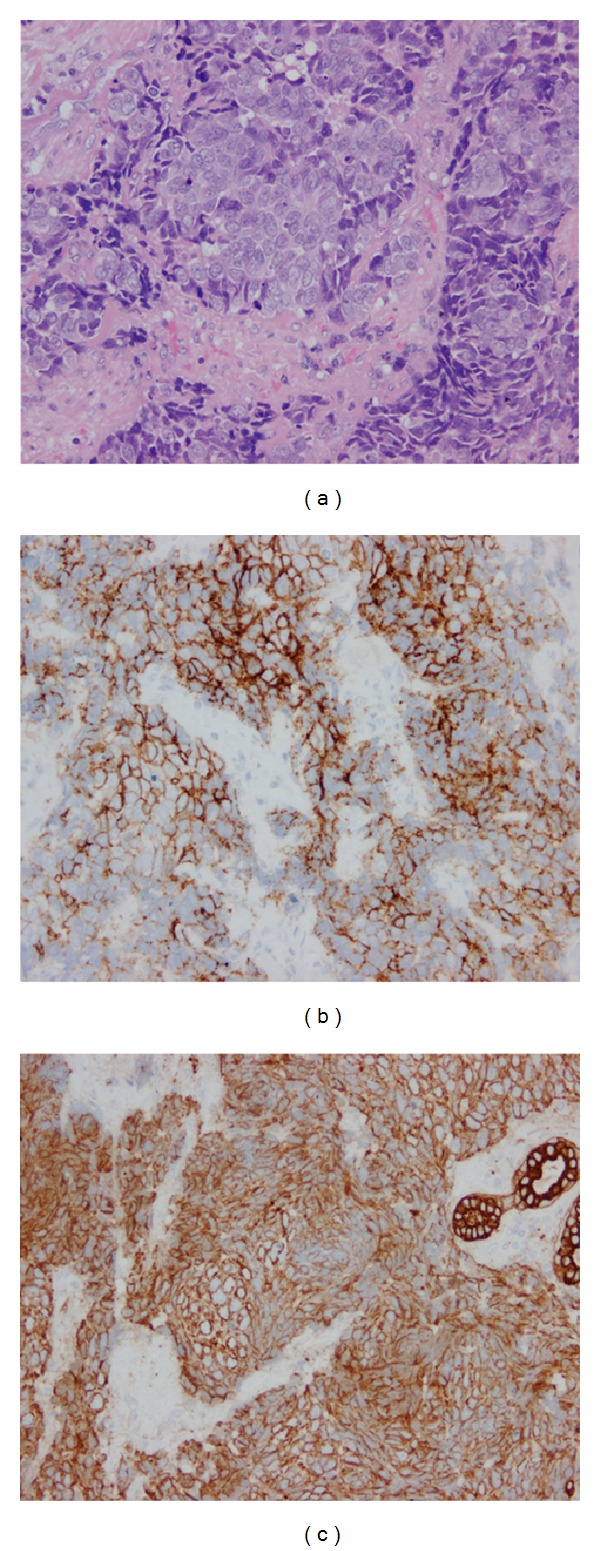
Representative histopathologic findings in the surgical specimen of the left lacrimal gland. H-E staining showed invasive proliferation of atypical small cells with rosette formation (a). Immunostaining with CD56 (b) and CK (AE1/AE3) (c) was positive (×200).

**Figure 2:**
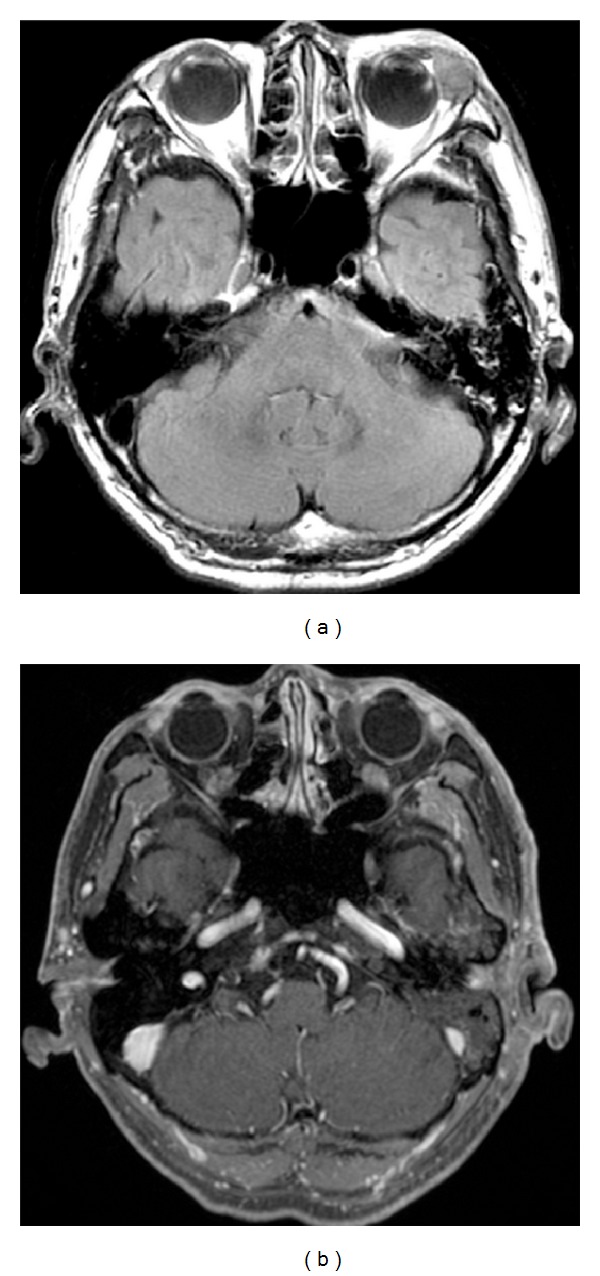
Pretreatment (a) and posttreatment (b) of MRI findings of the left lacrimal gland. After combination therapy, the tumor in the left lacrimal gland was not detectable.

An 86-year-old man noticed a swelling of his left upper eyelid three months earlier. Because the size of the nodule gradually increased, the patient was referred to the Chiba University Hospital. We performed excision biopsy, and histopathological examination indicated that the tissue had small atypical cells which were positive to CD56 and CK (AE1/AE3; Figure 1) but negative to TTF-1 and CK20. Magnetic resonance imaging (MRI; Figure 2) and positive emission tomography (PET) excluded systemic metastasis. The patient was diagnosed with a primary NEC of the left lacrimal gland. He underwent chemotherapy (carboplatin and etoposide) for four cycles, and the size of the tumor was significantly decreased. A month later, we performed tumor excision including the clinically visible margin, and a month later, the patient had 50 Gy radiotherapy of the surgical margins. After three-combination therapy, the tumor was undetectable in the MRI (Figure 2). A month later, however, a metastasis to the parotid lymph node was found, and the patient underwent total lymphadenectomy of the left parotid gland and 46 Gy radiotherapy was applied to the left neck. The patient had no recurrence for 6 months since the last radiotherapy. However very recently, the tumor was found to have metastasized to the liver, and he underwent chemotherapy for liver metastasis. Thus, a careful observation was still needed for this patient.

### 2.2. Case  2

**Figure 3:**
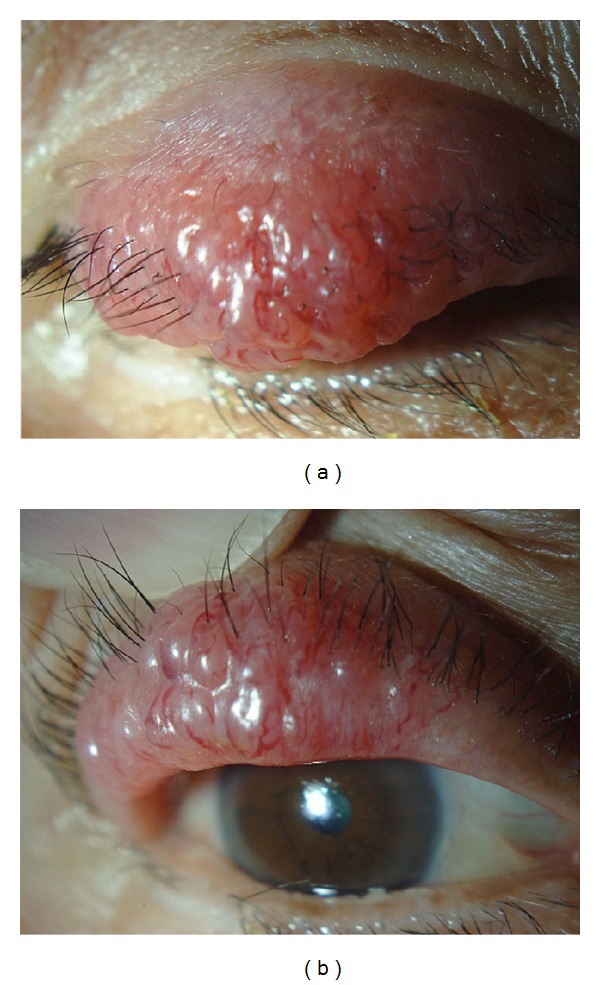
Clinical appearance of the right upper eyelid tumor. The painless, reddish, papillary, and solid 30 × 15 mm size nodule was observed in the right upper eyelid.

An 80-year-old man noticed a nodule of approximately 1 cm in the right upper eyelid and visited an ophthalmological clinic two weeks later. After 1 month of observation as a chalazion, the patient was referred to our hospital because the size was rapidly increasing. The findings on the first visit showed a painless, solid, reddish papillary mass of 30 × 15 mm size if the right upper eyelid (Figure 3). CT scans showed no other malignant tumors. Because of the rapid growth, a highly malignant tumor was suspected, and a complete surgical excision of the margins of the tumor was performed with histopathological confirmation of negative margins. 

**Figure 4:**
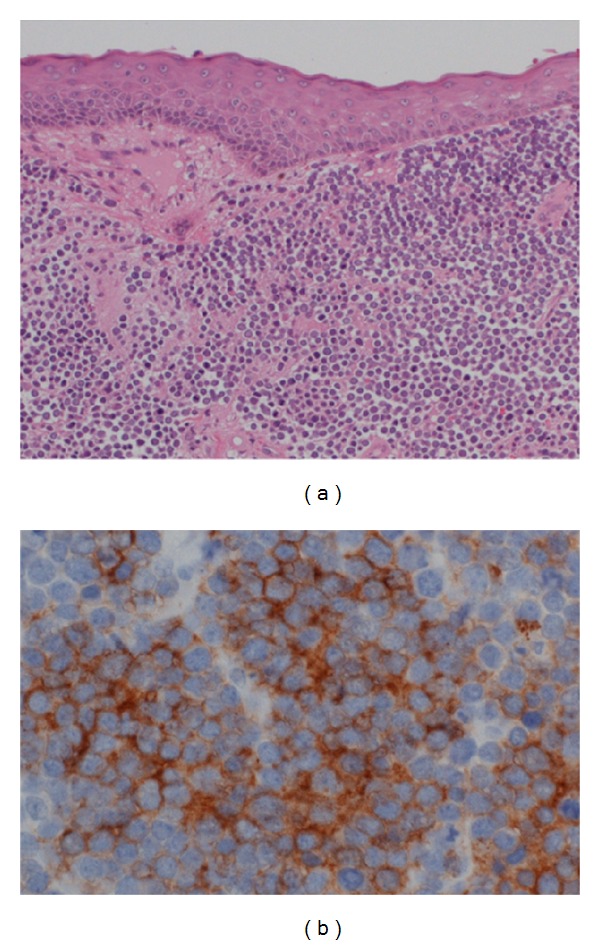
Representative histopathologic findings in the surgical specimen of the right upper eyelid. H-E staining showed small round cell tumor (a) (×100). Immunostaining with CD56 was positive (b) (×400).

Histopathological examination showed intradermal proliferation of small round tumor cells that were immunohistochemically positive to cytokeratin 20 with a distinct paranuclear dot-like quality, CD56, and chromogranin A but negative to LCA (Figure 4). Postoperative PET examination excluded systemic metastasis. The final diagnosis was a primary Merkel cell carcinoma of the right upper eyelid. Two weeks after surgery, he underwent 50 Gy radiotherapy on the neck and right side of the face to prevent metastasis. No recurrence or metastasis has been found for two years after the combination therapy.

## 3. Discussion

Primary small cell NECs of extrapulmonary sites are extremely rare [1], and Case  1 is the first report of primary small cell NEC of the lacrimal gland. The high ratio of Ki67-immunopositive cells (almost 100%) suggests that the tumor has high proliferative competence. Furthermore, the negative signals of immunostaining with TTF-1 suggest that it is unlikely to be a primary lung cell carcinoma. Two cases of primary Merkel cell carcinoma of the lacrimal gland have been reported [5, 6]; however, there is no established classification of NECs of the lacrimal gland. Thus, the histopathological diagnosis in this case strictly differentiated it from a Merkel cell carcinoma. 

On the other hand, Case  2 is a typical primary Merkel cell carcinoma of the upper eyelid but Merkel cell polyomavirus was negative in the tissue samples. Although Merkel cell carcinomas usually develop in elderly individuals or more frequently in immunocompromised patients, younger and healthy patients can also develop Merkel cell carcinoma in tissues that are Merkel cell polyomavirus positive [4, 6, 7]. Although Merkel cell polyomavirus was negative in the sample tissues of our case, we diagnosed the patient as having a typical Merkel cell carcinoma because of the elderly onset, the characteristic clinical features, and histopathological findings.

Neuroendocrine carcinomas and Merkel cell carcinomas are extremely rare but very aggressive, and tumors of >2 cm diameter have a poor prognosis [8]. However, there is no established therapy for the complete management of NECs because very few reports have been published especially for cases of NECs of the ocular adnexa. We have had patients with large cell NECs of the lung that metastasized to the iris leading to secondary glaucoma [9]. Because of the good visual acuity, we performed intravitreal bevacizumab injection and successfully improved the iris tumor and secondary glaucoma. Although we could improve the patient's quality of life, the patient died one month after the treatment because of the primary lung cancer [9].

Because lymph node metastasis is a sign of poor prognosis in patients with Merkel cell carcinoma [10], Case  2 underwent 50 Gy postoperative radiotherapy on the neck and on the right side of the face to try to prevent metastasis. The combination therapy seemed to be successful in our case. In the patient with NEC of the lacrimal gland (Case  1), the patient did not agree with a complete surgical excision by orbital exenteration. Thus, chemotherapy was performed followed by the surgical excision of the visible margins and postoperative radiotherapy. The tumor had metastasized to the neck lymph nodes, but after excision combined with radiotherapy, no recurrence has been detected during the follow-up period. However, liver metastasis has developed which may cause a poor prognosis. Although no information of prognosis in primary neuroendocrine carcinoma of ocular adnexa can be obtained, in sinonasal small cell neuroendocrine carcinoma, only 8% of patients were alive at 5 years [11]. An aggressive combined therapy is still required for these patients.

In conclusion, primary neuroendocrine carcinoma of the ocular adnexa is extremely rare but the tumor has high malignancy and readily metastasizes. Thus, combined therapy including surgery, radiotherapy, and chemotherapy is needed for complete management of neuroendocrine carcinomas.
